# Genome-wide analysis of MATE transporters and molecular characterization of aluminum resistance in *Populus*

**DOI:** 10.1093/jxb/erx370

**Published:** 2017-11-01

**Authors:** Nannan Li, Hongjun Meng, Haitao Xing, Lan Liang, Xin Zhao, Keming Luo

**Affiliations:** 1 Key Laboratory of Eco-environments of Three Gorges Reservoir Region, Ministry of Education, Chongqing Key Laboratory of Transgenic Plant and Safety Control, Institute of Resources Botany, School of Life Sciences, Southwest University, Chongqing, China; 2 College of Resources and Environment, Southwest University, Chongqing, China; 3 Academy of Agricultural Sciences, Southwest University, Chongqing, China

**Keywords:** Aluminum stress, citrate exudation, MATE transporter, *Populus*, root apex

## Abstract

Ionic aluminum (Al) in acidic soils, comprising approximately 50% of arable land globally, is highly toxic to most plant species. *Populus* grow naturally in acidic soils and tolerate high concentrations of Al. Multidrug and toxic compound extrusion (MATE) family genes in plants are involved in responses to Al tolerance. To date, however, the functional roles of the *MATE* genes in *Populus* remain unclear. In the present study, 71 putative MATE transporters were predicted in the genome of *Populus trichocarpa*. The chromosome distribution, phylogenetic relationships, and expression level analysis revealed that four candidate *MATE* genes belonging to subgroup IIIc might contribute to high Al tolerance in poplar. Further, the expression levels of two subgroup IIIc members, *PtrMATE1* and *PtrMATE2*, were induced by Al stress. Transient expression in onion epidermal cells showed that PtrMATE1 was localized to the plasma membrane. Overexpression of *PtrMATE1* increased Al-induced secretion of citrate from the root apex of transgenic plants. Al-induced inhibition of root growths were alleviated in both *PtrMATE1* overexpression lines in *Populus* and in Arabidopsis compared with wild-type plants. In addition, *PtrMATE1* expression was induced at 12 h after exposure to Al stress whereas *PtrMATE2* expression was induced at 24 h, indicating that these proteins coordinately function in response to Al stress in poplar. Taken together, these results provide important insights into the molecular mechanisms involved in Al tolerance in poplar.

## Teaser

Identification of all MATE family members in *Populus* and characterization of PtrMATE1 mediated citrate exudation from the roots of *Populus* in response to Al stress.

## Introduction

Aluminum (Al) toxicity is a major limitation to plant growth in acidic soils, namely with a pH<5.5, in which the Al complex in aluminosilicate clays is solubilized as the most toxic trivalent cation, Al^3+^ (Kochian *et al.*, 2004). Al^3+^ toxicity primarily targets the root apex, thereby inhibiting root growth (Liu *et al.*, 2009). In most plant species, two main types of Al resistance mechanisms have been described: Al exclusion mechanisms, which prevent Al from entering the root apex, and Al tolerance mechanisms, which detoxify and sequester Al in plants (reviewed by Kochian *et al.*, 2015). To date, the most well-characterized Al exclusion mechanism involves Al-induced exudation of organic acids (OAs), including malate, citrate, and oxalate efflux from roots (Delhaize and Craig, 1993; Delhaize and Ryan, 1995; Kinraide *et al.*, 2005; Delhaize *et al.*, 2007). These OA anions are transported from root cells into rhizospheres, in which Al^3+^ is effectively chelated, forming non-toxic complexes (Kinraide *et al.*, 2005). However, the regulatory mechanism of Al-induced oxalate secretion remains largely unknown (Feng *et al.*, 1998) and studies of functional genes related to the oxalate synthesis pathway did not reveal the regulation mechanism of oxalate excretion induced by Al in plants (Franceschi and Nakata, 2005; Xu and Peng, 2006).

Al^3+^ concentrations, even as low as 50 μM, affect root growth in herbaceous plants (Brauer, 2001; Kumari *et al.*, 2008; Chandran *et al.*, 2008). By contrast, woody plants generally tolerate high concentrations of Al and grow naturally in acidic soil (Schaedle *et al.*, 1989). For example, seedlings of Norway spruce (*Picea abies*) and birch (*Betula pendula*) showed no changes in root growth at Al concentrations ranging from 0.3 mM to 3 mM (Göransson and Eldhuset, 1987; Göransson and Eldhuset, 1991). Woody plants have evolved an adaptive mechanism that enables tolerance to high Al conditions (Grisel *et al.*, 2010). Elaboration of Al tolerance in woody plants will therefore broaden the current understanding of the Al tolerance mechanism in plants.

Currently, homologs of the malate transporter gene ALMT1 have been isolated from wheat (Sasaki *et al.*, 2004), Arabidopsis (Hoekenga *et al.*, 2006) and oilseed rape (Ligaba *et al.*, 2006). Many Al-induced citrate efflux genes have also been identified and characterized in herbaceous plants, which are all members of the MATE family, such as *HvAACT1* in barley (Furukawa *et al.*, 2007), *SbMATE* in sorghum (Magalhaes *et al.*, 2007), *AtMATE* in Arabidopsis (Liu *et al.*, 2009), *TaMATE* in wheat (Ryan *et al.*, 2009), *ScFRDL1* in rye (*Secale cereale* L.; Yokosho *et al.*, 2011), and *ZmMATE1* in maize (Maron *et al.*, 2010). The encoded proteins of all these homologous genes are primarily localized to root epidermis cells (Furukawa *et al.*, 2007) and are required for external Al resistance (Yokosho *et al.*, 2011). The MATE proteins are a large family of multidrug efflux transporters (Kuroda and Tsuchiya, 2009) that are widely distributed in plants, bacteria, fungi, and mammals (Omote *et al.*, 2006). Many putative MATE transporters have been predicted in Arabidopsis (Li *et al.*, 2002), *Oryza sativa* (Tiwari *et al.*, 2013), and *Medicago truncatula* (Zhao and Dixon, 2009). Recently, some plant MATE transporters have been implicated in controlling diverse developmental and physiological processes, including transport of secondary metabolites, such as alkaloids, flavonoids, and anthocyanins (Shoji *et al.*, 2009; Pérez-Díaz *et al*., 2014), detoxification of toxic compounds or heavy metals (Diener *et al.*, 2001; Li *et al.*, 2002), improvement of abiotic resistance (Sun *et al.*, 2011), and transport of phytohormones (Zhang *et al.*, 2014), iron (Yokosho *et al.*, 2009; Zhang *et al.*, 2014), and Al (Furukawa *et al.*, 2007; LiLiu *et al.*, 2009; Fujii *et al.*, 2012; Zhou *et al.*, 2013; Liu *et al.*, 2016). Screening the entire *MATE* gene family is therefore helpful to identify putative Al-induced citrate transporters from plant species.

Early phytoremediation studies showed that *Populus*, a fast-growing tree species, could be a suitable candidate for phytoremediation of metal-polluted soils (Cunningham and Ow, 1996; Migeon *et al.*, 2010). A recent study showed that *Populus* tolerates high concentrations of Al and that one of the aspen MATE homologous genes may be related to Al tolerance in the roots of aspen according to transcriptomic predictions (Grisel *et al.*, 2010) with the completion of the poplar genome sequence (Tuskan *et al.*, 2006). To date, however, the characterization of MATE genes in *Populus* remains lacking. In the present study, we performed a genome-wide search for all putative MATE transporters in *P. trichocarpa* according to whole genome sequence data from *P. trichocarpa* Torr. & A. Gray (http://genome.jgi-psf-org/Poptrl_l/Poptrl_l.home.html). The chromosome distribution, gene duplication, phylogenetic relationships, and gene and protein structures of these poplar putative MATE transporters were analyzed and the expression levels of 10 candidate *MATE* genes in the shoots and roots of *Populus* after Al treatment were characterized using qRT-PCR analysis. Furthermore, the function of *PtrMATE1* in transgenic poplar and Arabidopsis was identified and characterized. Finally, the coordinated roles of PtrMATE1 and PtrMATE2 in response to long-term Al stress were also investigated. These results provide insights into the Al tolerance mechanism in *Populus* under acidic soil conditions and will be helpful for further understanding the roles of *MATE* genes in *Populus*.

## Materials and methods

### Plant material and treatments

#### Plant materials and growth conditions

*P. trichocarpa* Torr. & A. Gray and *P. tomentosa* Carr. (clone 741) (Chinese white poplar) were cultivated in a greenhouse at 24^◦^C under a 14 h/10 h light/dark cycle with 5,000 lux of light and maintained in sterile woody plant medium (WPM) containing 0.8% (w/v) agar. The WPM medium contained the following macro- and micronutrients: NH_4_NO_3_ (400mg/L), Ca(NO_3_)_2_·4H_2_O (556mg/L), K_2_SO_4_ (990mg/L), CaCl_2_·2H_2_O (96mg/L), KH_2_PO_4_ (170mg/L), Na_2_MoO_4_·2H_2_O (0.25mg/L), MgSO_4_·7H_2_O (370mg/L), MnSO_4_·H_2_O (22.4mg/L), ZnSO_4_·7H_2_O (8.6mg/L), CuSO_4_·5H_2_O (0.25mg/L), FeSO_4_·7H_2_O (27.8mg/L), Na_2_-EDTA (37.3mg/L), vitamin B_1_ (1.0mg/L), vitamin B_6_ (0.5 mg/L), inositol (100 mg/L), nicotinic acid (0.5 mg/L), and glycine (2.0 mg/L) (Mccown and Lloyd, 1981). The normal pH was 5.2. In the present study, for the Al treatment experiments, the pH was 4.0. The plants were transplanted every 6–8 weeks.

#### Al and La treatments

In general, 4-week-old plant roots were immersed in WPM medium containing 500 µM AlCl_3_ or 500 µM LaCl_3_ at pH 4.0 during the treatment, and subsequently, the shoots and roots were collected and rinsed in WPM at pH 4.0. Plants treated with WPM medium, again at pH 4.0, were used as a control.

For the time-course experiments, 10 mm root apices were rinsed three times for 10 min each time with the 500 µM CaCl_2_ solution at pH 4.0 (Ca solution) to avoid potential ion leakage around root apices. The root apices were subsequently treated with a CaCl_2_ solution containing either 500 µM AlCl_3_ (Al solution) or 500 µM LaCl_3_ in 1.5 ml centrifuge tubes at 25^◦^C every 3h. The appropriate solutions and root samples were collected at each time point. Plants treated with the CaCl_2_ solution were used as controls. At least three biological replicates were performed for each treatment. All samples were immediately frozen in liquid nitrogen and stored at -80^◦^C until further analysis.

### Identification of MATE transporters in *Populus*

The genomic and protein sequences of 57 MATE transporters in Arabidopsis were obtained from Phytozome v11.0 (Goodstein *et al.*, 2012; http://phytozome.jgi.doe.gov/pz/portal.html). *P. trichocarpa* putative MATE protein sequences were retrieved by BLASTP searches against the target (*P. trichocarpa v3.0*) proteome in Phytozome v11.0 using the Arabidopsis MATE protein sequences as queries, with E-value≤1e-7). These MATE sequences were further filtered using Pfam (Marshall, 2003; http://pfam.xfam.org/) and the Simple Modular Architecture Research Tool (Letunic *et al.*, 2015; http://smart.embl-heidelberg.de/smart/batch.pl) based on the presence of a conserved MATE domain (Pfam: PF01554).

### Chromosomal locations and gene duplication analysis

The online tool PopGenIE (http://www.popgenie.org/) was used to determine and plot the chromosomal localization of all *PtrMATE* genes. Genes separated by five gene loci within a 100 kb distance were considered tandem duplicates (Hu *et al.*, 2010). Segmental duplications resulting from salicoid genome-wide duplications were identified based on the duplication coordinates from the *Populus* genome assembly. Blocks in the same colors represent the homologous chromosomal segments. Segmental and tandem duplication events of the MATE family were conducted according to Tuskan *et al.* (2006).

### Phylogenetic and structural analyses of MATE transporters in *Populus*

The full protein sequences of 71 *Populus* MATE proteins and 30 previously reported MATE proteins from other plant species (see Table S1 available at the Dryad Digital Repository http://dx.doi.org/10.5061/dryad.vb047) were used for multiple sequence alignments using ClustalW in MEGA 6.0 (Tamura *et al.*, 2013). The maximum likelihood (ML) was constructed by MEGA 6.0 using an algorithm with 1000 bootstraps, based on the equal input model, using partial deletion of 95% site coverage for gaps and missing data. The online program Gene Structure Display Server (GSDS) with default settings (Hu *et al.*, 2015; http://gsds.cbi.pku.edu.cn) was used to analysis the MATE gene structure. The online tool Multiple EM for Motif Elicitation (MEME; Bailey and Elkan, 1994; http://meme-suite.org/) was used to predict the motifs in MATE proteins. The maximum number of motifs was set at 10.

### RNA isolation and real-time quantitative PCR

Total RNA was extracted using the RNA RNeasy Plant Mini Kit (Qiagen, Duesseldorf, Germany). First-strand cDNA synthesis was performed using the PrimeScript™ RT reagent kit (Perfect Real Time; Takara, Dalian, China). GoTaq^®^ qPCR Master Mix (Promega, Madison, WI USA) was used to perform qRT-PCR to determine the transcript levels of *MATE* genes in *P. trichocarpa*. (See gene-specific primers used for qRT-PCR analysis in Table S2 at the Dryad). At least two biological replicates of each sample and three technical replicates of each biological replicate were performed to ensure the accuracy of the results. The reference gene UBQ (FJ438462) was used as an internal control. A relative quantification method was used to evaluate quantitative variation among replicates. The PCR conditions and relative gene expression calculations were conducted as previously described (Zhang *et al*., 2014).

### Citrate measurement

Citrate concentrations were determined based on the citric acid (CA) content of the test box (Jiancheng Bioengineering Institute, Nanjing, China) according to the manufacturer’s instructions. Briefly, citrate reduces Cr (VI) in acid solutions, and produces Cr^3+^, which has a specific absorption peak at 545 nm. By measuring the increase in absorbance at *A*_540_, the CA content in the sample can be calculated.

### Cloning of *PtrMATE1* and transformation of poplar and Arabidopsis

The full open-reading frame of *PtrMATE1* was amplified with gene-specific primers. The amplification products were cloned into the plant binary vector pCAMBIA1302. *Agrobacterium tumefaciens* strain GV3101 containing p*35S:PtrMATE1* was used to transform plants.

Poplar transformation was performed according to Jia *et al.* (2010). Briefly, poplar leaves were excised from *in vitro* plantlets, cut into disks, and dipped in the diluted Agrobacterium culture for 8–10min; the leaf disks were then transferred to WPM medium. The infected disks were co-cultivated in the dark for 2 d and subsequently transferred to callus-inducing medium. After 2–3 weeks, leaf disks with induced calli were subcultured on screening medium to induce adventitious buds. Regenerated shoots were transferred to rooting medium. The rooted plantlets were acclimatized in pots placed inside a humid chamber for 2 weeks and finally transferred to the greenhouse.

*A. tumefaciens* harboring the p*35S:PtrMATE1* construct was also transformed into wild-type Arabidopsis (Columbia ecotype, Col-0) and the T-DNA insertion mutants *AtMATE-KO* (SALK_081671; Liu *et al.*, 2009) by the floral dip method (Clough & Bent, 1998). Selection of transformants was performed on ½ Murashige and Skooge (MS) medium supplied with 50 mg·L^-1^ hygromycin. Homozygote progeny from T2 transformants with a single copy of the transgene were screened. The *PtrMATE1-OX* and *PtrMATE1-R* transgenic plants were found using qRT-PCR. *PtrMATE1-OX* was the overexpression of *PtrMATE1* in wild-type Arabidopsis, *PtrMATE1-R* was the overexpression of *PtrMATE1* in *AtMATE-KO* mutants.

### Evaluation of transgenic plants for resistance against Al stress

When the roots of wild-type and transgenic plants grew to 1 cm, the nutrient solution was replaced with autoclaved WPM medium at pH 4.0. After 2 d, the nutrient solution was replaced with a treatment solution comprising autoclaved WPM medium at pH 4.0, supplemented with AlCl_3_ up to 500 μM. The pH of the Al treatment solutions was adjusted to 4.0 with KOH.

Root growth was monitored photographically prior to the treatment at 2 d and during the entire treatment at 12 h intervals. We used a digital camera focused on a 0.1 mm grid of the graph paper. The pictures were cropped and normalized using the grid on the graph paper with IMAGEJ 1.50i. The normalized pictures were used to measure the increase in root length during particular time intervals. The root growth rate was estimated by dividing each increment by the time elapsed. Following these treatments, the roots were separated from the shoots and rinsed in WPM at pH 4.0. The root samples were collected and transferred to a sterile 1.5 ml tubes. The pooled leaves and stems were separately collected. All tissues were frozen in liquid nitrogen and stored at -80 °C until RNA extraction.

## Results

### Identification of *MATE* family genes in *Populus*

Genome-wide analysis was performed to predict all *MATE* genes in *P. trichocarpa* Torr. & A. Gray using genome sequence data (http://genome.jgi-psf-org/Poptrl_l/Poptrl_l.home.html). Initially, we identified 77 putative full-length protein sequences encoding *MATE* genes from the *Populus* whole genome using a BLASTP search with 57 MATE protein sequences in Arabidopsis (collected from Phytozome v11.0) as queries. These putative MATE sequences were filtered based on the presence of the conserved MATE domain (Pfam: PF01554) using the Pfam database. Eventually, a total of 71 genes encoding unique poplar *MATE* genes were identified and designated with consecutive nomemclature as *PtrDTX1-71* (for *Populus* detoxification 1-71) based on their physical location (Li *et al.*, 2002). These *MATE* genes encoded proteins that varied in length from 120 to 608 amino acids with an average of 480 amino acids, a molecular weight range of 13.14 to 65.49 kDa, and predicted isoelectric point values between 5.05 and 9.50. Detailed information for all 71 *Populus* MATE proteins is listed in Table S1 at Dryad.

### Chromosomal location and gene duplication of *Populus MATE* genes

**Fig. 1.**
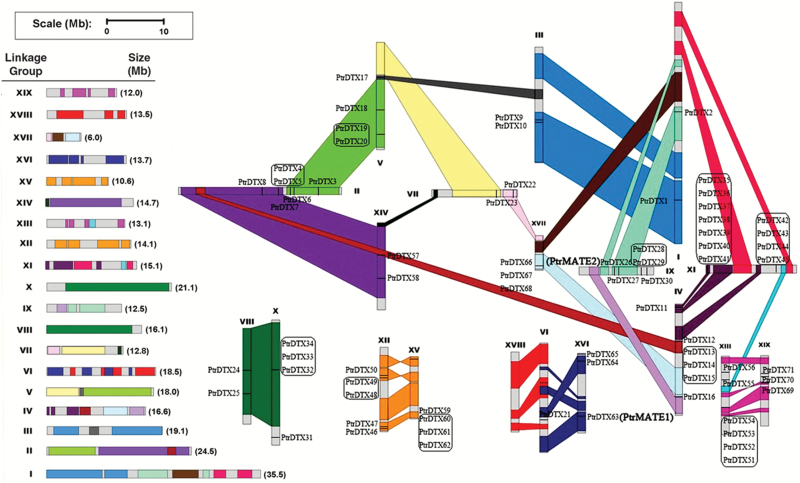
Chromosomal location of *Populus* MATE genes. A total of 71 MATE genes are mapped to the 18 linkage groups (LG). Schematic view of chromosome reorganization through recent whole genome duplication in *Populus* is shown. Segmentally duplicated homologous blocks are indicated with the same color. The scale represents megabases (Mb). The LG numbers are indicated at the top of each bar.

To examine the physical relationship of *PtrMATE* genes in the chromosomes of *Populus*, we performed chromosomal location and gene duplication analyses. As shown in Fig. 1, *in silico* mapping of the gene locus showed that 71 *Populus MATE* genes were mapped to 18 linkage groups (LG), except LG XVIII. LG XI harbored the highest number of *PtrMATE* genes at 11 genes, followed by LG II and LG XIII with 6 genes. Only one *PtrMATE* gene was observed on LG VI, and two *PtrMATE* genes were observed on each of LG I, III, VII, VIII, and XIV. Substantial clustering of *PtrMATE* genes was observed on LG XI (Fig. 1).

Recent studies have shown that the *Populus* genome has undergone at least three rounds of genome-wide duplications, followed by multiple segmental duplication, tandem duplication, and transposition events (Tuskan *et al.*, 2006; Hu *et al.*, 2012). The segmental duplication associated with a salicoid duplication event in particular contributed to the expansion of many multi-gene families (Barakat *et al.*, 2009; Rogers *et al.*, 2009; Hu *et al.*, 2010). We mapped *Populus MATE* genes to the duplicated blocks established in previous studies to determine the potential relationships between *MATE* genes and putative segmental duplications. The distributions of *MATE* genes relative to the corresponding duplicate blocks are illustrated in Fig. 1. Among these 71 genes, 36 genes were preferentially retained duplicates located in both duplicated regions and 18 duplicated blocks only contained *MATE* genes on one of the blocks and lacked duplicates on the corresponding block. Almost half of the genes, 35 of 71, were represented in distinct tandem duplicate gene clusters. These results revealed that tandem repeats and duplication events contributed to the expansion of the *MATE* gene family in the *Populus* genome.

### Phylogenetic analyses of PtrMATE proteins

**Fig. 2.**
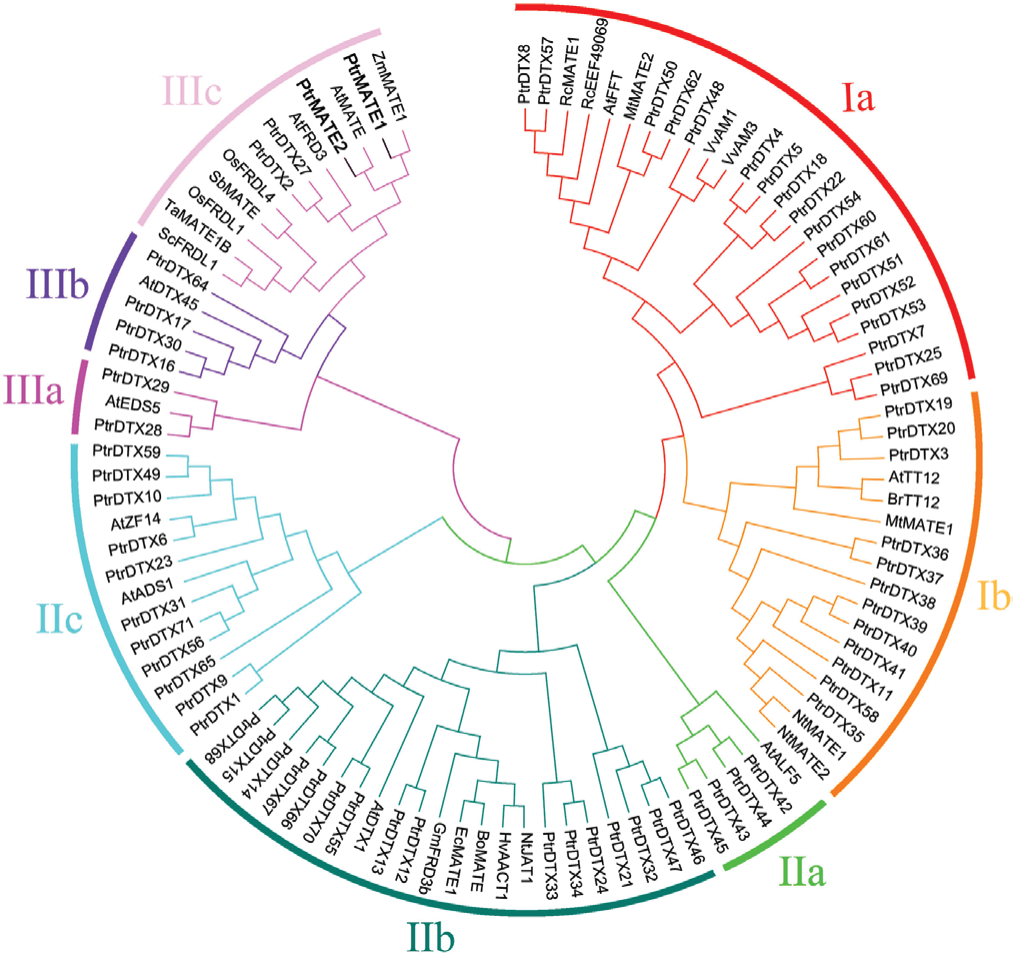
The phylogenetic tree of MATE proteins from *P. trichocarpa* and other plant species. The phylogenetic tree was constructed using MEGA 6.0 with the Maximum Likelihood (ML) method. Bootstrap values in percentages (1000 replicates) are indicated on the nodes. Different subgroups are highlighted using different colors and marked with arcs outside the cycle tree.

To characterize the phylogenetic relationships among *Populus* MATE proteins, we constructed a phylogenetic tree using the full-length proteins of the 71 *Populus* MATE members and 30 previously reported plant MATE proteins from 15 other plant species using Clustal W and MEGA 6.0 (Fig. 2; Table S1 at Dryad). These PtrMATE proteins can be classified into three subfamilies with eight smaller subgroups, namely subfamily I (subgroups Ia and Ib), II (subgroups IIa, IIb, and IIc), and III (subgroups IIIa, IIIb and IIIc). The functions of the PtrMATE proteins were inferred from functionally characterized MATE proteins from other plant species according to their phylogenetic relationships.

Subfamily I contains 24 transporters in subgroup Ia and 17 in subgroup Ib. Among these proteins, many MATE members have previously been implicated in the transport of flavonoids, alkaloids, and other phenolics in Arabidopsis, *Medicago truncatula*, grapevine, and tobacco (Zhao and Dixon, 2009; Shoji *et al.*, 2009; Gomez *et al.*, 2009; Thompson *et al.*, 2010; Gomez *et al.*, 2011), suggesting that this MATE subfamily might be involved in the transport and accumulation of secondary metabolites. There are three subgroups in subfamily II: IIa, IIb and IIc. The IIa subgroup comprised four PtrMATE proteins and AtALF5, which confers Arabidopsis resistance to toxins (Diener *et al.*, 2001). Subgroup IIb, comprised 16 PtrMATEs and six characterized MATE proteins involved in the efflux of plant-derived antibiotics and other toxic compounds, and detoxification of heavy metals, among other functions (Li *et al.*, 2002; Morita *et al.*, 2009). In addition, subgroup IIc includes 11 PtrMATEs and AtADS1 and AtZF14. Interestingly, the functions of AtADS1 and AtZF14 are diverse, including the regulation of plant disease resistance, increasing the leaf initiation rate, and the regulation of hypocotyl cell elongation and iron homeostasis (Sun *et al.*, 2011; Seo *et al.*, 2012; Zhang *et al.*, 2014; Wang *et al.*, 2015). However, most of these identified CA exporters belong to subfamily III. These transporters secrete OAs to the apoplast to chelate Al^3+^ in the rhizosphere and alleviate Al^3+^ toxicity in acidic soils. At least eight known MATE transporters, including ScFDRL1, TaMATE1B, OsFRDL1, SbMATE, OsFRDL4, AtFRD3, AtMATE, and ZmMATE1, have been associated with Al detoxification and/or iron translocation (Yokosho *et al.*, 2009; Tovkach *et al.*, 2009; Liu *et al.*, 2009; Maron *et al.*, 2010; Sivaguru *et al*., 2013; Charlier *et al.*, 2015). Subgroup IIIc also contains four *Populus* members, PtrMATE1, PtrMATE2, PtrDTX2, and PtrDTX27, suggesting that these PtrMATEs might also be involved in Al detoxification or iron translocation in *Populus*.

### Structure and motif distribution analysis of *Populus* MATE genes

**Fig. 3.**
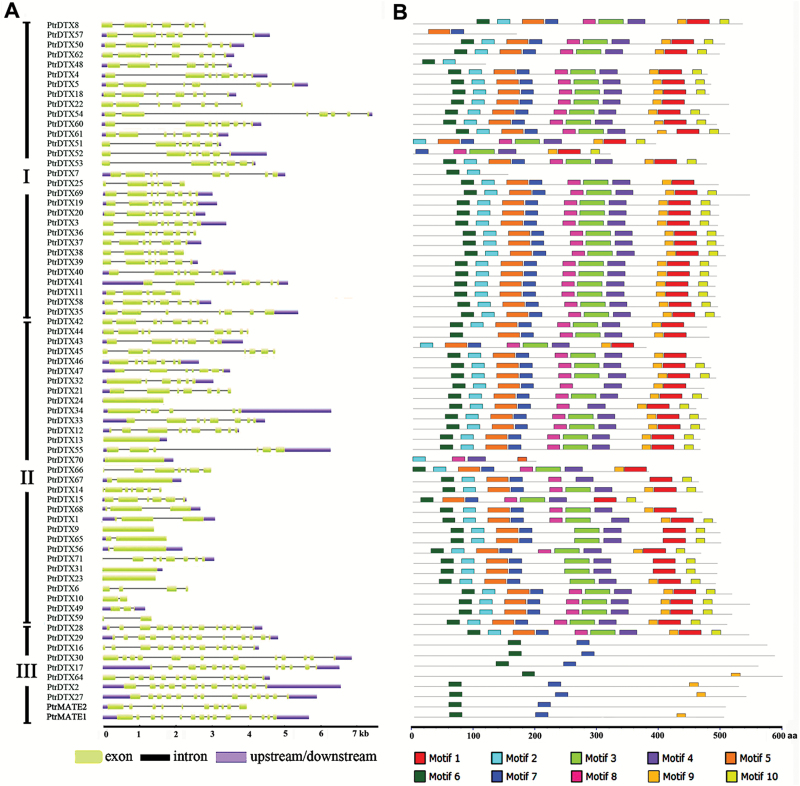
The gene structures and conserved motifs of PtrMATE family members. (A) Exons and introns of *PtrMATE* genes are plotted using green boxes and black lines, respectively. The blue boxes indicate upstream/downstream sequences. (B) Protein motifs of the PtrMATE family. The motifs of *Populus* MATE proteins are shown as colored boxes, each motif is represented as a number in the colored box. The genes are listed according to the order of subfamily I to III from the phylogenetic tree and different subfamilies are highlighted with lines.

To better understand their functional characteristics, the structural diversity of all of the *Populus MATE* genes was analyzed by comparing the corresponding coding sequences with their genomic DNA sequences. The *PtrMATE* genes contained 1–14 exons, and these intron-exon structures are plotted in Fig. 3A. Consistent with the characteristics shown in the phylogenetic analysis, closely related genes were more structurally similar, differing in the lengths of introns and exons in the same subfamily. For example, almost all of the *PtrMATE* genes in subfamily I contained seven exons and the members in subfamily II had one (PtrDTX23, PtrDTX31) to nine (PtrDTX12) exons, while the *PtrMATE* genes in subfamily III exhibited the largest number of exons, between 10 to 14.

We also predicted conservative motifs in PtrMATE proteins using MEME software. A total of 10 conserved motifs were identified as shown in Fig. 3B and Table S3 at Dryad. The types and sequences of the motifs were similar among subfamilies I and II but significantly different from subfamily III. Fewer motifs were observed among subfamily III proteins compared with the other subfamily members. All of the MATE family members contained motif 3 except for PtrDTX10 (Fig. 3B).

### Expression patterns of the *PtrMATE* genes in response to Al stress

**Fig. 4.**
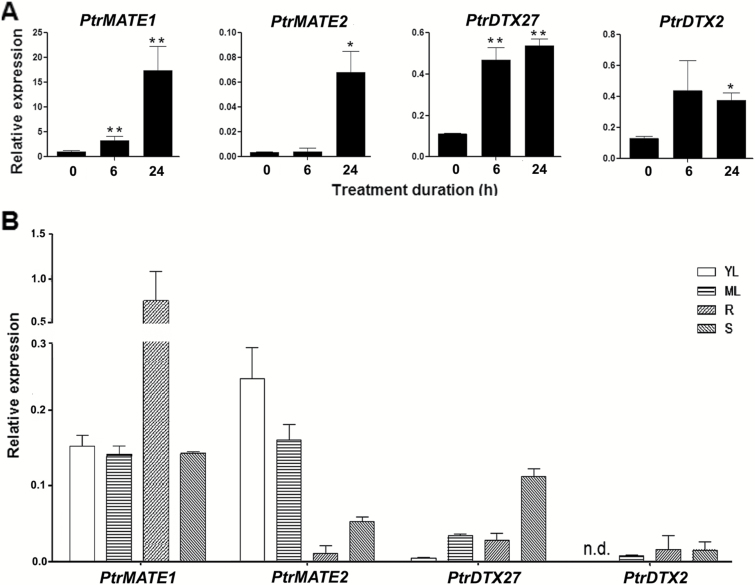
Expression analysis of subgroup III *MATE* genes using qRT-PCR. (A) Expression of four *PtrMATE* members in poplar roots under 500 μM Al^3+^ for 6 h and 24 h. (B) Relative quantities of four members in roots, stems, young leaves, and mature leaves are illustrated. Expression of *PtrMATE1* in wild-type roots was arbitrarily fixed at one. The results are shown as the mean expression ± standard deviation (SD) of three independent experiments. Poplar *ubiquitin* (*UBQ*) expression was used as a control and gene-specific primers were used for qRT-PCR analysis of *PtrMATE* genes. Student’s *t*-test, *P<0.05, **P<0.01.

To investigate the function of the *PtrMATE* genes in subfamily III, we determined the expression patterns of these genes in response to Al exposure using quantitative real-time PCR (qRT-PCR) with the 4-week-old poplars immersed in the WPM medium containing 500 µM Al^3+^ at pH 4.0) for 12 h. Poplar *ubiquitin* (*UBQ*) expression was used as a control and gene-specific primers were used for qRT-PCR analysis of *PtrMATE* genes. The results revealed that the expression levels of all of the subgroup IIIc genes were significantly increased in roots treated with 500 μM Al for 12 h, while other subgroup III members were not induced (see Fig. S1 at Dryad). A time-course experiment showed that *PtrMATE1* was activated 6 h after the exposure to Al and at 24 h the expression increased by approximately 18 times compared with the level detected prior to Al treatment (Fig. 4A). Both *PtrDXT2* and *PtrDXT27* were also upregulated at 6 h under Al stress conditions. By contrast, *PtrMATE2* was not induced at 6 h after Al treatment but was significantly upregulated at 24 h. These results suggest that these *PtrMATE* genes might be involved in the response to Al stress.

We also examined the expression patterns of these four subgroup IIIc genes in different tissues of *Populus*, including the roots, stems, young leaves, and mature leaves. As shown in Fig. 4B, these *PtrMATE* genes had different tissue-specific expression patterns. *PtrMATE1* was highly expressed in the roots and its expression was more abundant than that of other *PtrMATE* genes, suggesting that PtrMATE1 might play a critical role in response to Al stress in poplar.

### Sequence analysis and subcellular localization of *PtrMATE1*

To investigate the function of *PtrMATE1* in response to Al stress, we further focused on the characterization and analysis of *PtrMATE1* in *Populus*. In the *Populus MATE* gene family, *PtrMATE1* and *PtrMATE2* shared 64% amino acid sequence identity with each other; these genes are the closest sequences to AtMATE, a citrate transporter, with 65% and 66% identity, respectively. Multiple alignments of PtrMATE1 and PtrMATE2 with the AtMATE protein also revealed that they share strong sequence homology with each other (see Fig. S2 at Dryad). Consistent with the structure of *AtMATE*, *PtrMATE1* and *PtrMATE2* were predicted to have similar topology using the CCTOP program (Liu *et al.*, 2009; http://cctop.enzim.ttk.mta.hu/).

**Fig. 5.**
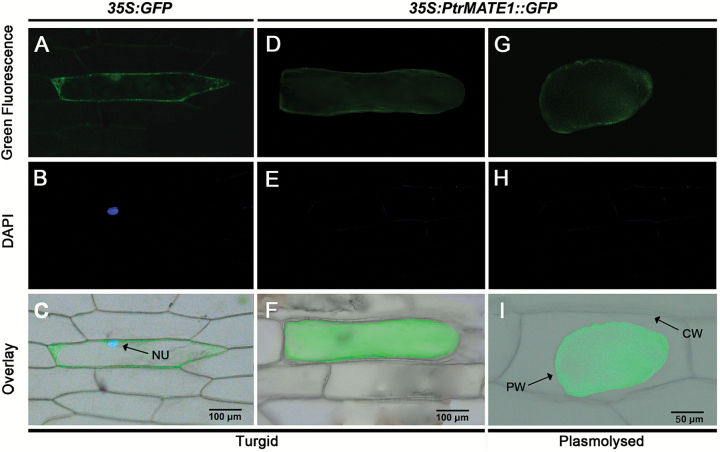
Subcellular localization of *PtrMATE1*. GFP alone (A–C) or fusion protein PtrMATE1::GFP (D–F) were transiently expressed in onion epidermal cells, respectively. The images were acquired before (D–F) and after (G–I) plasmolysing the cells with 0.1 M sucrose. The overlay images of the brightfield and fluorescence images are shown (C, F, I). Scale bar, 100 μm (C, F) or 50 μm (I). NU, nucleus; PM, plasma membrane; CW, cell wall.

To determine the subcellular location of PtrMATE1, the p*35S:PtrMATE1-GFP* construct, in which the *PtrMATE1-GFP* fusion gene was under the control of the *CaMV 35S* promoter, was transformed into onion epidermal cells using Agrobacterium-mediated transformation. The PtrMATE1-GFP signal was observed in the plasma membrane of onion epidermal cells (Fig. 5D, E, F), compared with the control that expressed GFP alone (Fig. 5A, B, C). To distinguish localization in the plasma membrane from that in the cell wall, we added 0.1 M sucrose to induce plasmolysis. In plasmolyzed cells, the GFP signal was exclusively detected in the plasma membrane after transformation of the p*35S:PtrMATE1-GFP* construct (Fig. 5G, H, I), indicating that *PtrMATE1* is localized to the plasma membrane, consistent with other citrate permeable plant MATE homologs.

### Expression patterns of *PtrMATE1*

**Fig. 6.**
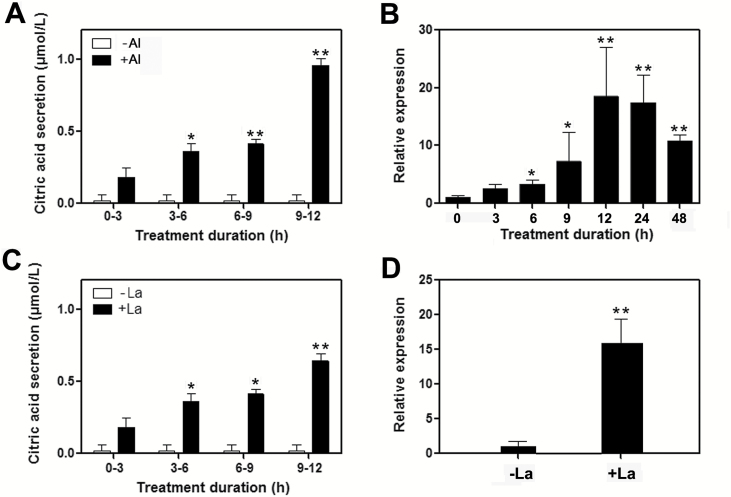
Citrate release and *PtrMATE1* expression in poplar roots in response to Al and La treatments. (A) Induction of citrate secretion from the root apices of poplar in response to Al treatment. (B) Time course of *PtrMATE1* expression in the roots of poplar in response to Al treatment. (C) Induction of citrate secretion from root apices of poplar in response to La treatment. (D) *PtrMATE1* expression in the roots of poplar in response to 12 h La treatment. Excised root apices, 10 mm in length, were placed in the solution containing 0 μM or 500 μM Al or La, respectively. Data are presented as the means ± standard deviation (SD) of three independent experiments. Poplar *ubiquitin* expression was used as a control. The results are shown as the mean expression ± standard deviation (SD) of three independent experiments. Student’s *t*-test, *P<0.05, **P<0.01.

There are two different OA secretion patterns in response to Al treatment based on the timing of secretion in plants (Ma *et al.*, 2001). To establish the OA release pattern of *Populus* and the connection between citrate and gene inducible secretion, we performed a time-course analysis of citrate secretion and *PtrMATE1* expression in the root apex of poplar. The citrate secretion rates remained unchanged for the duration of the treatment without Al stress. However, with Al treatment, citrate secretion increased at 3 h, continued to increase, and at 9-12 h the secretion rate rapidly increased (Fig. 6A). In parallel, the transcript abundance of *PtrMATE1* increased with Al treatment, reached a peak at 12 h and subsequently gradually decreased (Fig. 6B). These results indicated that *Populus* shows the typical pattern of OA release in response to Al exposure.

Several studies have shown that several lanthanides such as La^3+^, have ionic properties similar to Al^3+^ (Kataoka *et al.*, 2002; Liu *et al.*, 2013) but citrate secretion in rice bean specifically occurs in response to Al stress not La stress (Liu *et al.*, 2013). To determine whether both citrate secretion and *PtrMATE1* expression in *Populus* were specific to Al stress, we analyzed *PtrMATE1* expression and citrate secretion under La treatment. The results showed that La enhanced the expression of *PtrMATE1* in *Populus* (Fig. 6D) and that La-induced secretion of citrate from the roots was also increased (Fig. 6C), although the expression level and citrate secretion were relatively lower in La-stressed roots than that in Al-stressed roots. Interestingly, *PtrMATE2* expression was not induced after 12 h of La treatment (see Fig. S3B at Dryad).

**Fig. 7.**
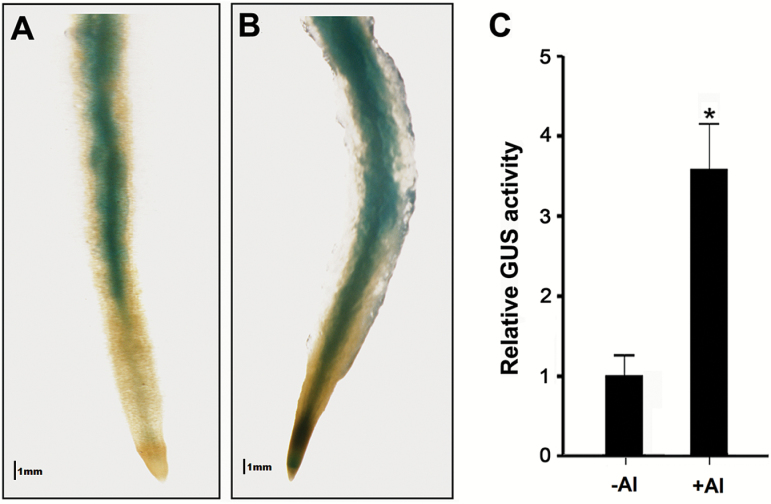
GUS activity in roots of transgenic *PtrMATE1p::GUS* plants. The *PtrMATE1* gene promoter-driven GUS expression vector was introduced into *P. tomentosa* Carr. Transgenic plant seedlings were treated with 500 μM Al^3+^ for 0 h (A) and 12 h (B), respectively. GUS staining was observed in the mature roots of transgenic poplar. Scale bars, 1 mm. (C) Quantitative GUS activity in the roots of transgenic plants. Student’s *t*-test, *P<0.05. Results are shown as mean expression ± standard deviation (SD) of three independent experiments.

To further confirm the expression pattern of *PtrMATE1*, the 2 kb promoter fragment of *PtrMATE1* was fused to a GUS reporter gene and transformed into wild-type poplar. GUS staining was observed in the central cylinder of mature roots but not detected in the root tips without Al (Fig. 7A). After exposure to Al, GUS activity was not only observed in the central cylinder but also extended to the entire root apex (Fig. 7B). GUS activity was quantitatively measured using a F-7000 fluorospectrophotometer (Hitachi, Japan) with fluorospectrophotometry (Jefferson *et al.*, 1987). The results showed the GUS activity in the 2 cm root apices was increased by approximately 3.5 times under Al stress conditions (Fig. 7C).

### Overexpression of *PtrMATE1* in transgenic poplar confers citrate efflux and Al tolerance

**Fig. 8.**
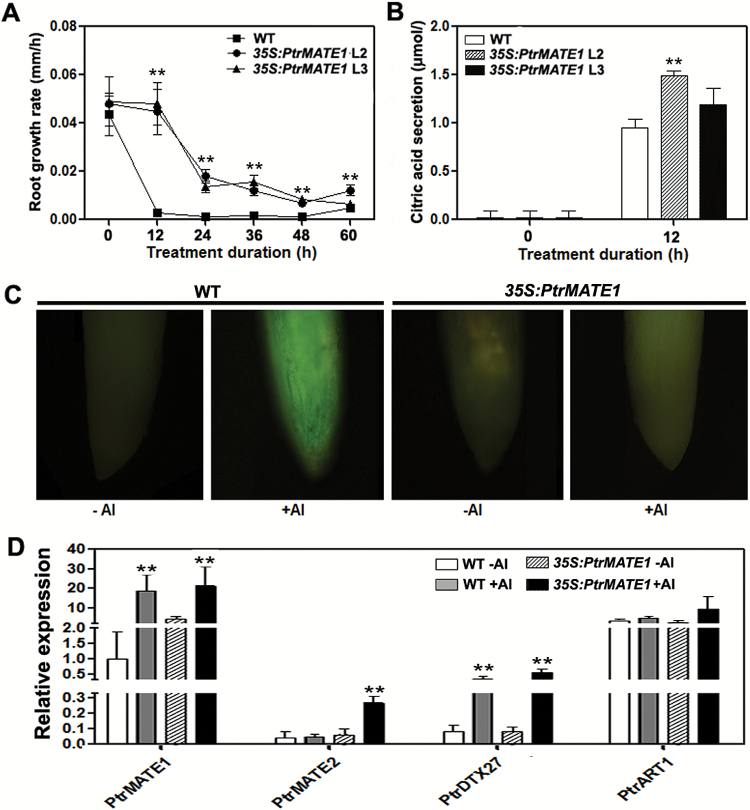
Overexpression of *PtrMATE1* in transgenic poplar confers citrate efflux and Al tolerance. (A) Root growth of wild-type and transgenic plants overexpressing *PtrMATE1* in nutrient solution containing 500 μM Al^3+^ for 60 h. All results represent the means ±standard deviation (SD) of three independent experiments. (B) Induction of citrate secretion from root apices of wild-type and transgenic poplar in response to 500 μM Al^3+^ treatment for 12 h. Data are represented as the means ± standard deviation (SD) of three independent experiments. (C) Callose accumulation in the root tips following Al treatment. Poplar seedlings were exposed to a nutrient solution containing 0 M (-Al^3+^) or 500 μM AlCl_3_ (+Al^3+^) for 12 h. Seedlings were subsequently fixed, stained with 0.1% aniline blue at pH 9.0, and observed using fluorescence microscopy. The fluorescence images indicate callose accumulation. WT-Al^3+^, wild-type plants in a solution without Al^3+^; WT+Al^3+^, wild-type plants in a solution containing 500 μM Al^3+^; *35S:PtrMATE1*-Al^3+^, transgenic plants in a solution without Al^3+^; *35S:PtrMATE1*+Al^3+^, transgenic plants in a solution containing 500 μM Al^3+^. (D) Relative expression levels of *PtrMATE1*, *PtrMATE2*, *PtrDTX27*, and *PtrART1* in roots of wild-type and transgenic *35S:PtrMATE1* plants under 500 μM Al^3+^ treatment for 0 h and 12 h. Student’s *t*-test, **P<0.01.

To further investigate the function of *PtrMATE1* in mediating Al transport, transgenic poplar plants overexpressing *PtrMATE1*, named *35S::PrtMATE1*, were generated and grown in the greenhouse. The transcript levels of *PtrMATE1* were determined in these transgenic lines using qRT-PCR (see Fig. S4 at Dryad) and two independent lines, L2 and L3, with high *PtrMATE1* expression were selected for further analysis. We first compared Al tolerance between wild-type and transgenic plants. A time-course experiment showed that, at 12 h after the exposure to Al, strong inhibition of root growth was observed in wild-type, with a reduction in growth rate of ≥90% (Fig. 8A). By contrast, the root growth rate of the transgenic lines, L2 and L3, was only reduced by 10% and 15% at the same time point, respectively. Up to 48 h under Al treatment, the root growth rates of the transgenic lines were significantly higher than that of the control (*P*<0.01). Furthermore, citrate secretion in transgenic poplar overexpressing *PtrMATE1* was enhanced in the presence of Al compared with the wild-type control (Fig. 8B). This *in vivo* result consistently indicated that PrtMATE1 is a citrate transporter induced by Al stress in poplar. Additionally, the *PtrMATE1* gene driven by the 35S promoter was also transformed into the wild-type and *AtMATE* knockout mutant, *AtMATE-KO*, Arabidopsis to generate the overexpression lines, named *PtrMATE1-OX*, and the functional complementation line, named *PtrMATE1-R*, respectively. Overexpression of *PtrMATE1* in Arabidopsis strengthened the response to Al stress (Fig. 6S at Dryad). The results also indicated that *PtrMATE1* complements the function of *AtMATE* in response to Al stress.

To further confirm that *PtrMATE1* correlated with Al tolerance in *Populus*, we detected the callose content in root apex stained with aniline blue, which is an indicator of Al toxicity and accumulates in plants upon Al exposure (Kochian *et al.*, 2005). In the absence of Al, the root apex of both wild-type and transgenic poplar exhibited a weak fluorescence signal (Fig. 8C). Intense fluorescence, indicating callose accumulation, was observed in the root tips of wild-type plants under Al stress conditions. By contrast, even in the presence of Al, callose deposition in the root tips of the *PtrMATE1* overexpression lines was slightly higher than in the untreated control. Additionally, with Al treatment, *PtrMATE2* expression was also upregulated in the *PtrMATE1* overexpression lines, but the transcript level of *PtrART1* (Al resistance transcription factor 1, Yamaji *et al.*, 2009) was not changed (Fig. 8D). This result indicated that Al toxicity was significantly ameliorated in the *PtrMATE1* overexpression lines and further confirmed that PrtMATE1 is a citrate exporter in response to Al stress in poplar.

### Functional coordination of PtrMATE1 and PrtMATE2 in response to long-term exposure to Al

**Fig. 9.**
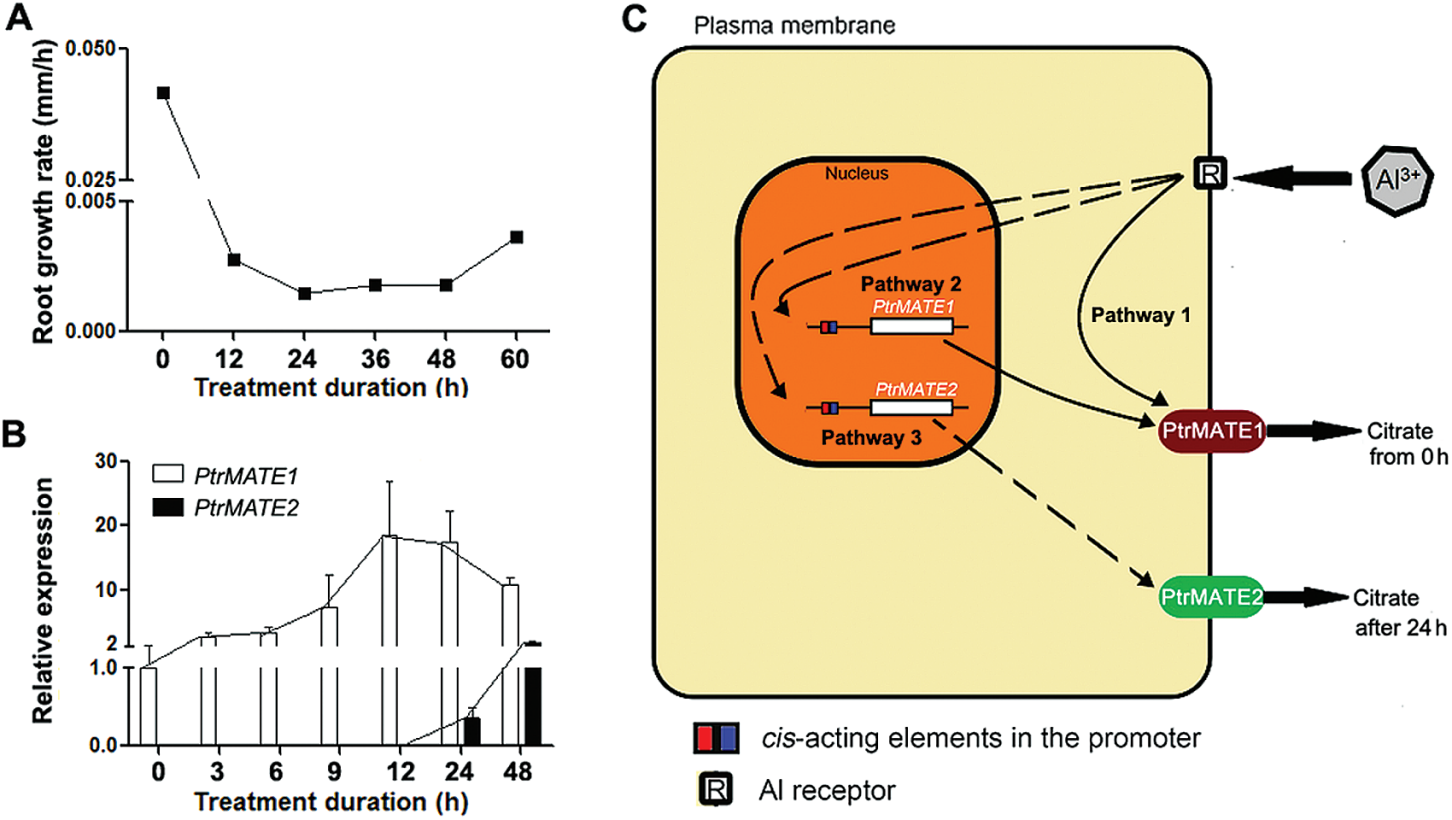
Hypothetical model for PtrMATE transporter-mediated citrate secretion in response to Al stress in roots of *Populus.* (A) Root growth of wild-type plants in nutrient solution containing 500 μM Al^3+^ for 60 h. (B) Expression levels of *PtrMATE1* and *PtrMATE2* in poplar roots during 48 h under Al treatment. Expression of the poplar *ubiquitin* (*UBQ*) gene was used as a control. All results are shown as the mean expression ± standard deviation (SD) of three independent experiments. (C) Hypothetical model for Al-induced citrate secretion from roots of *Populus*. There are at least three pathways involved in the process. Pathway 1: the receptor (R) on the plasma membrane binds Al^3+^ and activates *PtrMATE1* to transport citrate immediately out of the cell through the plasma membrane. Pathway 2: *PtrMATE1* expression is induced by Al^3+^ and subsequently PtrMATE1 transports citrate out of the cell. Pathway 3: *PtrMATE2* expression is induced by Al^3+^ at 24 h after treatment and subsequently PtrMATE2 transports citrate in cooperation with PtrMATE1.

Phylogenetic analysis of MATE proteins showed that Arabidopsis AtMATE corresponds to PtrMATE1 and PtrMATE2 in *Populus* (Fig. 2). To determine the functional diversity of these duplicated pairs in response to Al stress, we analyzed the relationship between the root growth rate of *Populus* and expression levels of *PtrMATE1* and *PtrMATE2* under Al stress. The results showed that, although the transcript level of *PrtMATE1* was decreased at 24 h after Al treatment (Fig. 9B), the root growth rate was stable between 24 h to 48 h during Al treatment (Fig. 9A). Time-course analysis of *PtrMATE2* expression during the 48 h Al treatment showed that the transcript levels of *PtrMATE2* increased 10 times at 24 h and 150 times at 48 h after exposure to Al, compared with the untreated control (Fig. 9B; Fig. S3A at Dryad). The different expression patterns of *PtrMATE1* and *PtrMATE2* suggested functional diversification and potential function redundancy in Al tolerance in poplar.

## Discussion

### Characterization of the *Populus MATE* gene family

In the present study, a total of 71 *MATE* genes, classified into three subfamilies, were identified in the *P. trichocarpa* genome (Fig. 2). Each subfamily contains at least one MATE motif (Fig. 3). The lengths of the *Populus* MATE proteins, comprising 120 to 608 amino acids, were significantly varied, whereas in Arabidopsis, the lengths of the MATE proteins ranged from 400 to 700 amino acids (Li *et al.*, 2002). These *PtrMATE* genes displayed marked variation in gene structure and protein motifs, implying a high degree of complexity among the *Populus* MATE family. The integration and realignment of the gene fragments induced exon-intron increases or decreases (Xu *et al.*, 2012). Gene structure variations therefore play a major role in the evolution of gene families (Xu *et al.*, 2012). In addition, the number of exons in the *PtrMATE* genes divided into phylogenetic groups were nearly the same, but varied between different subfamilies. Obviously, the *PtrMATE* genes in subfamily III contained more exons than the other members (Fig. 3).

Previous studies have shown that the *Populus* genome undergoes at least three rounds of genome duplication, followed by multiple segmental duplications, tandem duplications, and transposition events (Tuskan *et al.*, 2006; Hu *et al.*, 2012). The segmental duplications associated with salicoid duplication events in particular contributed to the expansion of many multi-gene families (Barakat *et al.*, 2009; Rogers *et al.*, 2009; Hu *et al.*, 2010). In the present study, almost 80% of the *PtrMATE* genes were located in duplicated regions and approximately half of the *PtrMATE* members were represented in distinct tandem duplicate gene clusters (Fig. 1). These results suggest that the expansion of the *PtrMATE* gene family in the *Populus* genome largely reflected duplication events and tandem repeats. We also observed that most of the Arabidopsis *MATE* genes have at least two pairs of orthologous genes in the *Populus* genome (Fig. 2). The loss of a few *MATE* orthologous genes in poplar might reflect dynamic rearrangements following segmental duplication (Li *et al.*, 2015).

### PtrMATE proteins respond to Al stress under acidic conditions

Approximately 50% of the potential arable soil worldwide is acidic soil, in which Al toxicity is one of the major factors limiting plant growth and crop yields (reviewed by Kochian *et al.*, 2015). Al-induced citrate and malate exclusion are the two major Al resistance processes in both dicots and monocots, mediated by members of the MATE and ALMT families, respectively (Kochian *et al.*, 2015). In recent decades, many Al-induced citrate transporter genes of the MATE family have been identified and characterized, including *SbMATE* from sorghum (Magalhaes *et al.*, 2007), *HvMATE* from barley (Furukawa *et al.*, 2007), *AtMATE* from Arabidopsis (Liu *et al.*, 2009), *VuMATE* from rice bean (Yang *et al.*, 2011) and *ZmMATE* from maize (Maron *et al.*, 2010). Interestingly, many tree species grow normally in acidic soil and often exhibit natural tolerance to Al toxicity. Previous studies have shown that aspen (*Populus tremula*) tolerates high concentrations of Al by releasing citrate and oxalate from roots in response to Al stress (Grisel *et al.*, 2010). In this biological process, the citrate efflux transporter gene *MATE* is an important component of the Al tolerance mechanism in aspen.

To elucidate the molecular mechanism of Al tolerance in poplar, in the present study, we isolated an Al-inducible MATE gene *PtrMATE1* from *P. trichocarpa.* As shown in Fig. S2 (available at Dryad), PtrMATE1 with a conserved transmembrane domain structure shares 65% amino acid identity with AtMATE, a major contributor to Arabidopsis Al tolerance (Liu *et al.*, 2009). *PtrMATE1* expression was detected after exposure to Al stress, while citrate exudation from roots could also be detected as early as 3 h after Al exposure (Fig. 6). GUS staining showed that *PtrMATE1* expression was specifically high in the central cylinder of mature roots of poplar and was significantly increased under Al treatment (Fig. 7). Overexpression of *PtrMATE1* in *Populus* resulted in an associated increase in Al tolerance and root citrate exudation (Fig. 8). Notably, the timing of *PtrMATE1* upregulation by Al is closely correlated with the onset of Al-induced citrate exudation from roots. This rapid regulation of *PtrMATE1* expression and citrate exudation by Al is consistent with that in maize, but different from that in sorghum and rice bean (Magalhaes *et al.*, 2007; Maron *et al.*, 2010; Yang *et al.*, 2011).

Although the functional and structural characteristics of PtrMATE1 are similar to those of reported citrate-permeable MATE proteins, the mechanism for Al resistance mediated by PtrMATE1 in poplar is different from other MATEs in herbaceous plants. First, in Arabidopsis and crop plants, Al concentrations as low as 50 µM are capable of inhibiting root growth (Kumari *et al.*, 2008; Chandran *et al.*, 2008), whereas poplar plants grow normally in acidic soil with high Al concentrations of 200 µM (Qin *et al*., 2007), implying that *Populus* has developed adaptive mechanisms to tolerate high Al stress. Most citrate-permeable *MATE* genes from other plant species are activated in response to a low Al concentration of 50 µM (Yokosho *et al.*, 2009; 2011; Wang *et al*., 2015). However, expression of *PtrMATE1* was induced by 500 µM and even 1000 µM Al treatment (Figs 4A and 7). These results confirmed that PtrMATE1-mediated citrate secretion is responsible for resistance to high Al concentration stress in poplar. Also transformed into Arabidopsis *Atmate* knockout mutants was *35S:PtrMATE1* and here we observed that PtrMATE1 strengthened the activity of Al-induced citrate exudation in transgenic Arabidopsis (see Fig. S6 at Dryad). Second, expression of *PrtMATE1* remained relatively constant, peaking at 12–24 h after exposure to Al (Fig. 6), a period almost two times longer than that of other MATE homologs in rice bean and maize (Maron *et al.*, 2010; Liu *et al.*, 2013). Third, *PtrMATE1* expression is not only upregulated by Al stress but also highly induced by La^3+^ treatment (Fig. 6D). Unlike in rice bean, citrate secretion specifically occurs in response to Al stress but not La stress (Liu *et al.*, 2013). These differences suggest that the citrate exudation response in *Populus* and herbaceous species may be regulated by different mechanisms. Additionally, ART1, a C2H2-type zinc finger transcription factor in rice, regulates expression of the MATE gene *OsFRDL2* in Al tolerance (Yamaji *et al.*, 2009; Yokosho *et al*., 2011). Thus, high Al tolerance is achieved through an ART1-regulated pathway in rice. However, *PtrART1* expression was not regulated by Al stress in wild-type or transgenic *35S:PtrMATE1* poplar plants (Fig. 8D), suggesting that other unknown components, such as transcription factors, might be involved in the response to Al in *Populus*.

### Coordinated roles of PtrMATE1 and PtrMATE2 enhanced resistance to long-term Al stress

Previous studies have shown that the *Populus* genome has undergone genome-wide duplications, followed by multiple segmental and tandem duplications (Tuskan *et al.*, 2006). Phylogenetic analysis showed that the Arabidopsis *AtMATE* gene corresponds to *PtrMATE1* and *PtrMATE2* in *Populus* (Fig. 2). Interestingly, *PtrMATE1* expression was induced by Al, peaking at 12 h after Al treatment (Fig. 6B), whereas the expression level of *PtrMATE2* was low before 24 h of Al exposure, but rapidly upregulated after 24–48 h (Fig. S3 at Dryad). The root growth rate of *Populus* was rapidly reduced at 12 h after Al exposure, but remained constant between 12–48 h. We therefore speculated that PtrMATE1 might play a major role in Al-induced citrate exudation from poplar roots at the early stage and that PtrMATE1 and PtrMATE2 coordinately mediate citrate release after 24 h of Al exposure. Future studies should include a more detailed analysis of PtrMATE2 function to clarify the mechanism underlying PtrMATE-mediated exudation of citrate in poplar roots in response to Al stress.

Finally, we propose a hypothetical model for Al-induced citrate secretion from roots of *Populus*. As illustrated in Fig. 9C, there should be at least three different pathways mediating citrate efflux in response to Al stress in *Populus*. In pathway 1, the Al-stress signal receptor (R) directly activates PtrMATE1 to export citrate out of the roots without post-transcriptional or translational level regulation. As shown in Fig. 6A, CA was detected in the root in response to treatment with Al. In pathway 2, transcriptional factor(s) or upstream control elements are synthesized *de novo* after sensing the receptor and further regulate the expression and translation of *PtrMATE1*. Subsequently PtrMATE1 continues to transport citrate out of the cell. In Fig. 6A, after Al treatment, expression of PtrMATE1 and CA secretion both generally increased with time. In pathway 3, *PtrMATE2* expression was induced after 24 h of Al treatment; PtrMATE2 may transport citrate in cooperation with PtrMATE1 (Fig. 9C). As described above, the expression level of *PrtMATE2* was significantly upregulated after Al treatment for 24 h, while *PtrMATE1* expression smoothly decreased (Figs 9A, B and S3A). Taken together, these results not only reveal the functional diversity of PtrMATE transporters in *Populus* against Al stress but also provide insights into Al tolerance mechanisms in *Populus* grown in acidic soil. The present study will be helpful to enhance the current understanding of the roles of *MATE* genes in *Populus* and also provides an important resource for the generation of tree or crop varieties more suitable for growth on acidic soils.

## Data deposition

The following data are available at Dryad Data Repository: http://dx.doi.org/10.5061/dryad.vb047

Sequences of all MATE proteins from *Populus*, Arabidopsis, rice, and 13 plant species.

## Supplementary Data

Supplementary data are available at *JXB* online.

Fig. S1. Expression of 10 PtrMATE genes in poplar shoots and roots under 500 μM Al3+ for 12 h.

Fig. S2. Multiple sequence alignment of PtrMATE1, PtrMATE2 and AtMATE.

Fig. S3. Time course of PtrMATE2 expression in the roots of Populus in response to Al or La treatment.

Fig. S4. qRT-PCR analysis of transgenic poplar plants.

Fig. S5. PtrMATE1 expression in the roots of poplar under Al treatments with different concentration.

Fig. S6. Overexpression of PtrMATE1 in transgenic AtMATE knockout mutant (ATMATE-KO) and wild-type Arabidopsis confers Al tolerance.

Table S1. Details of MATEs from different species.

Table S2. Primers used for qRT-PCR and gene cloning in the present study.

Table S3. Motifs of PtrMATE proteins.

## Supplementary Material

TABLE. S1

TABLE. S2

TABLE. S3

Fig.S1

Fig.S2

Fig.S3

Fig.S4

Fig.S5

Fig.S6

## Abbreviations:

Al: aluminium
CA: citric acid
MATE: multidrug and toxic compound extrusion
OAs: organic acids
PtrDTX1-71: *Populus* detoxification 1-71
WPM: woody plant medium.
