# Supplementary figures and images for: Genome-wide analysis of MATE transporters and molecular characterization of aluminum resistance in Populus
Source: J Exp Bot. 2017 Nov 1;68(20):5669–83. doi: 10.1093/jxb/erx370 (PMC5853298; doi:10.1093/jxb/erx370)

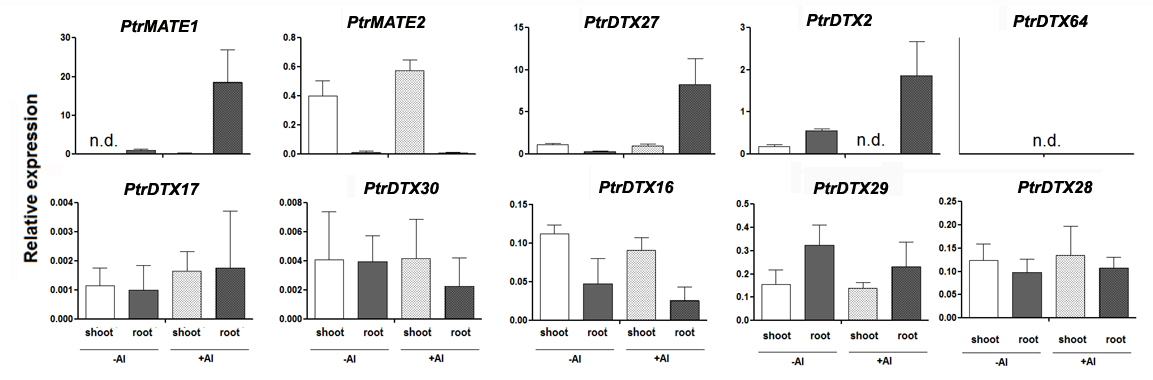

Supplement: Fig.S1 [file erx370_suppl_fig.s1.png]

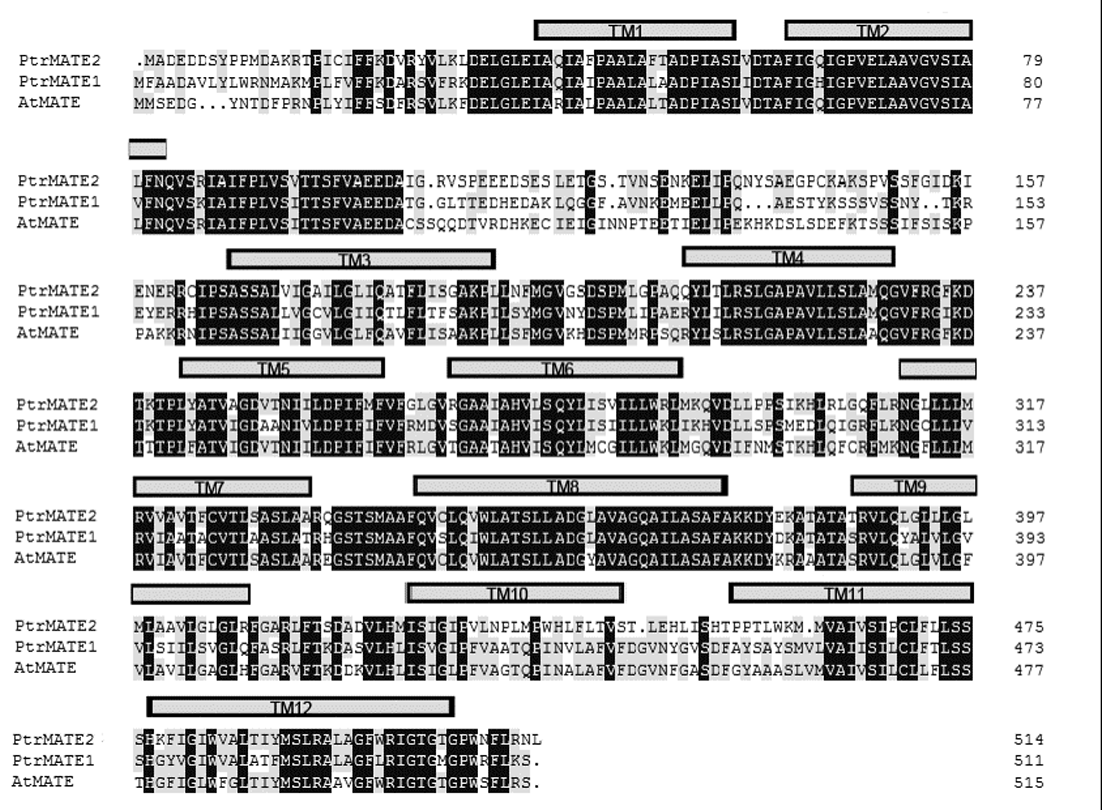

Supplement: Fig.S2 [file erx370_suppl_fig.s2.png]

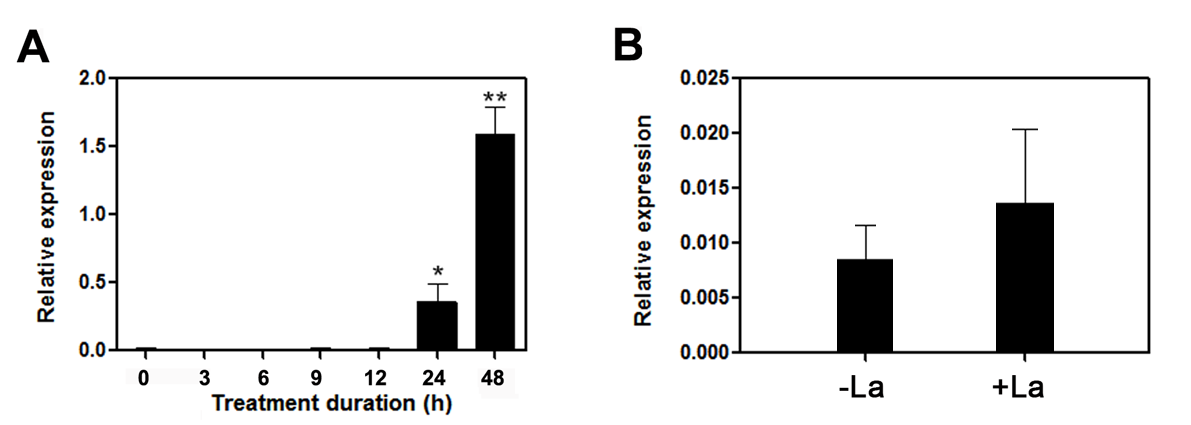

Supplement: Fig.S3 [file erx370_suppl_fig.s3.png]

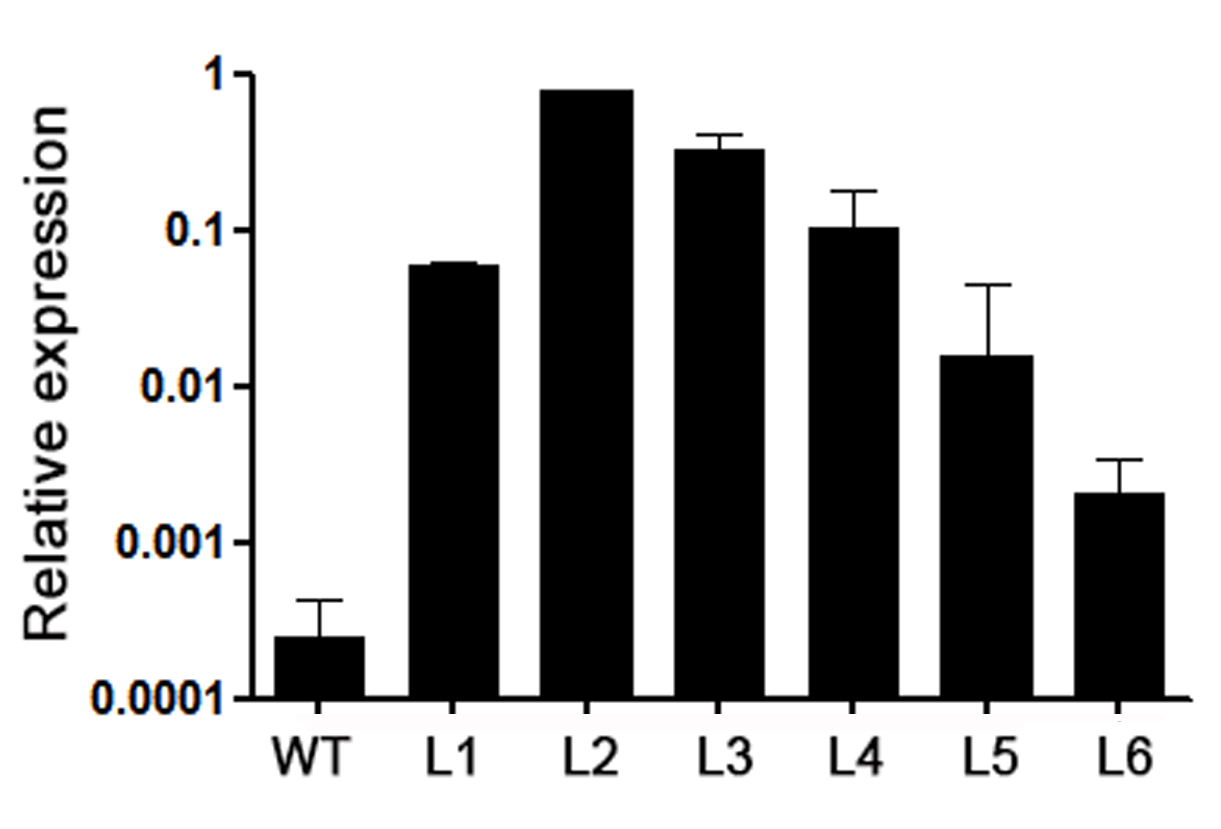

Supplement: Fig.S4 [file erx370_suppl_fig.s4.png]

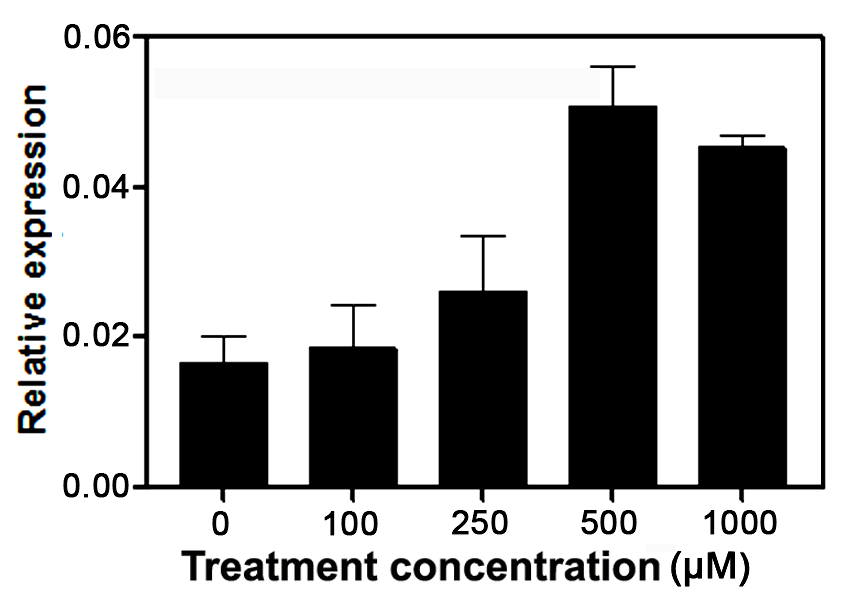

Supplement: Fig.S5 [file erx370_suppl_fig.s5.png]

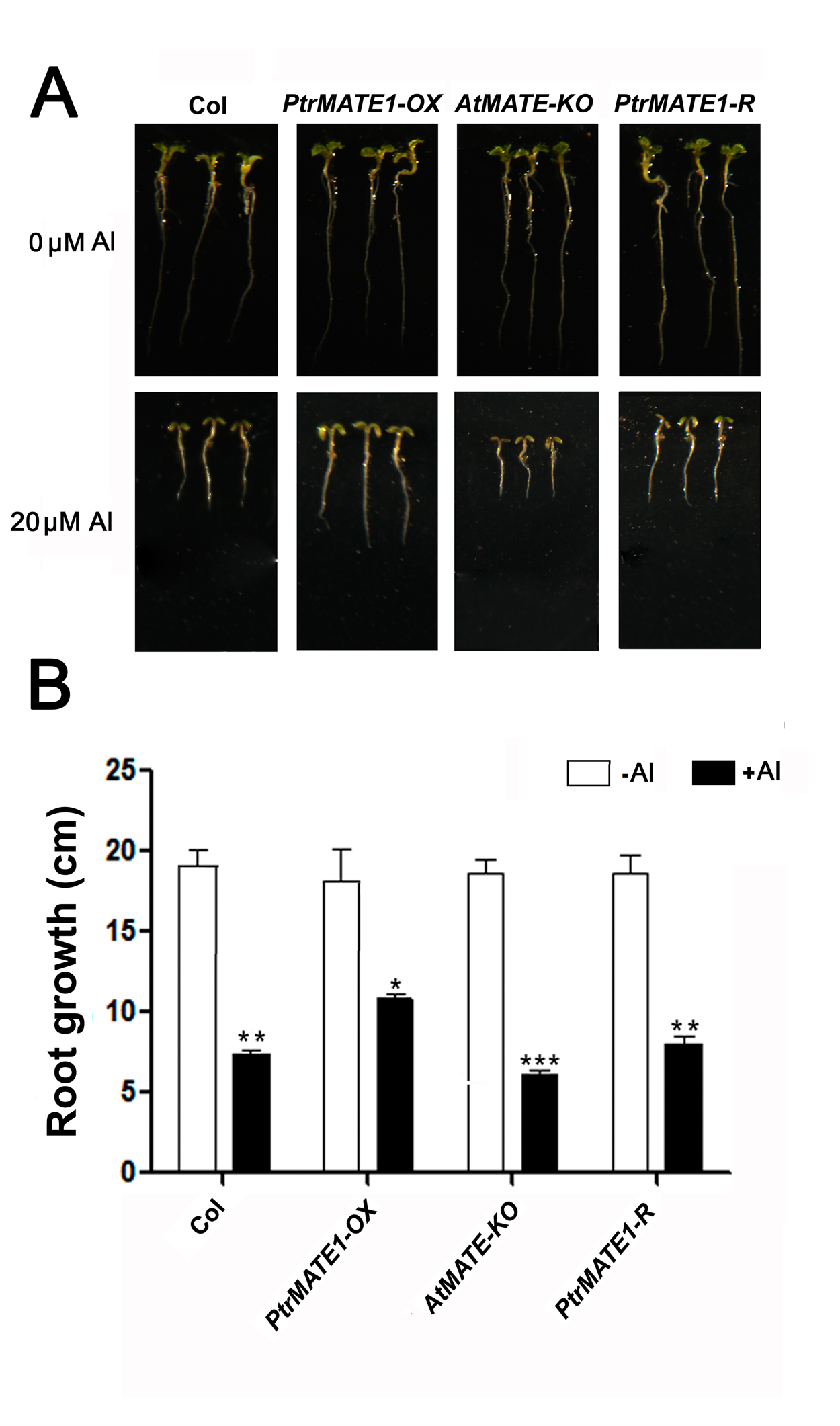

Supplement: Fig.S6 [file erx370_suppl_fig.s6.png]
